# The relationship between clinical outcomes of arthroscopic rotator cuff repair and hemoglobin A1c

**DOI:** 10.1016/j.asmart.2022.09.004

**Published:** 2022-10-05

**Authors:** Ryosuke Takahashi, Yukihiro Kajita, Yusuke Iwahori, Yohei Harada

**Affiliations:** a Department of Orthopaedic Surgery, Ichinomiya Nishi Hospital, Japan; b Department of Orthopaedic Surgery, Asahi Hospital, Japan; c Department of Orthopaedic Surgery, Hiroshima University, Japan

**Keywords:** Arthroscopic rotator cuff repair, Hemoglobin A1c

## Abstract

**Background:**

The clinical outcomes of diabetic patients who underwent arthroscopic rotator cuff repair have been reported. However, few studies have focused on the relationship between these clinical outcomes and postoperative hemoglobin A1c. This study compared clinical outcomes of diabetic patients with a hemoglobin A1c < 7% to those with a hemoglobin A1c ≥ 7% at 12 months post-arthroscopic rotator cuff repair.

**Methods:**

This retrospective study involved 51 consecutive patients who underwent arthroscopic rotator cuff repair from 2015 to 2020. Approximately 26 and 25 shoulders were assigned to a Low group (hemoglobin A1c < 7%) and High group (hemoglobin A1c ≥ 7%) at 12 months postoperatively, respectively. Patients with poorly controlled diabetes were preoperatively hospitalized for perioperative diabetic control. Clinical outcomes were shoulder range-of-motion, Constant Shoulder Score, and University of California, Los Angeles Score preoperatively and at 12 months postoperatively. Complications were also evaluated.

**Results:**

Both groups showed significant improvement in their range-of-motion, Constant Shoulder Score, and University of California, Los Angeles score at 12 months post-arthroscopic rotator cuff repair (p < 0.05) except external rotation in High group. These improvements were significantly inferior in the High group compared to the Low group (Low/High group; 167.3 ± 7.7°/159.5 ± 16.3° for forward flexion; p = 0.013, 94.9 ± 7.6/90.1 ± 9.9 points for Constant Shoulder Score; p = 0.033, 32.6 ± 3.6/30.6 ± 4.1 points for University of California, Los Angeles score, p = 0.037). Hemoglobin A1c at 12 months postoperatively was also significantly inferior in the High group (Low/High group; 6.5 ± 0.3%/7.6 ± 0.8%; p < 0.05). The rate of rotator cuff retear was not significantly different between groups (Sugaya type4; p = 0.49, type5; p = 0.322) and there were no cases of infection or shoulder stiffness in either group.

**Conclusion:**

Diabetic patients showed improvement of their range-of-motion and function after arthroscopic rotator cuff repair; however, patients with poorly controlled diabetes had significantly inferior improvement.

## 1 Introduction

Rotator cuff tear is a common shoulder disorder with a prevalence ranging from 13% in patients aged >50 years to >50% in those aged >80 years.^1^ Two-thirds of these lesions are asymptomatic, usually owing to their small size, whereas one-third are accompanied by symptoms such as pain, muscle weakness, and limited range-of-motion (ROM).^1^^,^^2^ For these individuals, a rotator cuff tear may lead to restrictions in their daily activities. Surgical repair of the rotator cuff using arthroscopy can be a potential solution in cases of refractory status as a conservative treatment. Arthroscopic rotator cuff repair (ARCR) is considered an exceedingly safe and effective procedure in appropriately indicated patients. However, complications such as infection, shoulder stiffness, and retear do occur.^3^, ^4^, ^5^

Diabetes mellitus is one of the most common and debilitating medical conditions. Long-term effects of uncontrolled hyperglycemia include peripheral neuropathy, kidney dysfunction, immunodeficiency, retinopathy, and musculoskeletal and tissue-repair disorders.^6^ Patients with diabetes may have increased surgical risks including infection, compromised tissue quality for repair, and a propensity of problems with wound healing.^6^^,^^7^ Furthermore, sustained hyperglycemia may increase the possibility of anatomic failure at the repaired cuff.^6^

The role of hemoglobin A1c (HbA1c), a serologic marker for average glucose amount in the bloodstream over a two to three-month period, has expanded in the diagnosis and treatment of diabetes.^8^ In addition to the enhanced risk of complications associated with the diagnosis of diabetes mellitus, multiple studies have linked hyperglycemia with increased rates of adverse outcomes in a variety of conditions, often irrespective of a diagnosis of diabetes mellitus.^9^^,^^10^ Elevated HbA1c has also been shown to be a risk factor in inferior clinical outcomes and complications.^3^^,^^11^^,^^12^

Several authors have reported the relationship between diabetes mellitus and ARCR.^13^, ^14^, ^15^, ^16^ Previous studies have investigated the association of perioperative glycemic control with clinical outcomes and complications after ARCR.^3^^,^^6^^,^^13^^,^^16^ However, to the best of our knowledge, no studies have focused on the relationship between clinical outcomes of ARCR and postoperative HbA1c. This study aimed to compare the clinical outcomes of ARCR in diabetic patients with an HbA1c < 7% to those with an HbA1c ≥ 7% 12 months post-ARCR.

## 2 Materials and methods

### 2.1 Study participants

We retrospectively reviewed the outcome of ARCR in two groups of patients, diabetic patients with an HbA1c < 7% and those with an HbA1c ≥ 7% 12 months post-ARCR. ARCR was performed on 51 shoulders in 51 patients in our hospital from April 2015 to May 2020. Each patient gave informed consent to participate in this institutional review board-approved study. A total of 26 and 25 shoulders were assigned to Low group (HbA1c < 7%) and High group (HbA1c ≥ 7%), respectively. We measured the longitudinal and transverse dimensions of the tear on the preoperative magnetic resonance imaging (MRI) along the oblique coronal plane and oblique sagittal plane, respectively.^17^ Tear size was categorized as small (<1 cm), medium (1–3 cm), large (3–5 cm), or massive (>5 cm) according to Cofield et al.^2^ Patients with shoulder osteoarthritis, calcified tendinitis, instability, hemiplegia after stroke, bone metastasis in the shoulder region, history of shoulder fractures, or history of shoulder surgeries were excluded. Preoperative HbA1c levels were measured 3 weeks before surgery to confirm diabetic status or euglycemic phenotype and to quantify the severity of sustained hyperglycemia before surgery. Diabetic patients with a preoperative HbA1c ≥ 7% were referred to as uncontrolled patients, and those with a preoperative HbA1c <7% were referred to as controlled patients.^6^^,^^16^ In our hospital, uncontrolled diabetic patients were preoperatively hospitalized for 1 week before surgery and underwent perioperative diabetic control.

We evaluated shoulder ROM (forward flexion, external rotation, and internal rotation at 0° of abduction), Constant Shoulder Score, and University of California, Los Angeles (UCLA) score preoperatively and 12 months after ARCR. We also evaluated complications (the rate of rotator cuff retear, stiff shoulder, and infection). We defined stiff shoulder as limited shoulder ROM in three directions, i.e. passive forward flexion of ≤100°, passive external rotation at the side of ≤10°, and internal rotation of ≤ L5.^18^ Internal rotation was defined as the highest vertebral body that the patient was able to reach with the thumb of the affected arm. Internal rotation was scored as follows: above T12: 6 points; above L5: 4 points; buttock: 2 points; and below buttock: 0 points. MRI was performed preoperatively and at 12 months after surgery. Repair integrity after ARCR was classified into 5 categories according to the Sugaya classification using oblique coronal, oblique sagittal, and transverse views of T2-weighted images.^19^ Using this classification system, types 4 and 5 were considered to be retears.

### 2.2 Surgical technique

All operations were performed uniformly under general anesthesia and in a beach chair position by a single skilled surgeon (Y.K.). For stiff shoulder, manipulation was performed before arthroscopy. In shoulders resistant to manual manipulation, arthroscopic capsular release was performed. A standard ARCR was performed using suture anchors and all rotator cuff tears were repaired using a Healix Transtend™ Implant System (Healix BR; DepuySynthes Co., Raynham, MA, USA) loaded with 3 high-strength sutures (Versalok Anchor; Depuy-Mitek Inc., Raynham, MA, USA). The number of anchors was decided according to the size of the tear and repair configuration in terms of the suture-bridge repair. Tenotomy or tenodesis was performed in case of a biceps long head lesion. All patients were administered 1 g acetaminophen intravenously every 6 h for 24 h postoperatively. This repeated dose regimen was chosen based on previously published trials that confirmed its safety and efficacy.^20^

### 2.3 Postoperative protocol

The postoperative protocol was identical in both groups. The shoulders were immobilized for 4 weeks with an abduction brace. Isometric rotator cuff exercises and passive exercises for forward flexion and relaxation of the muscles around the shoulder girdle were initiated on the day after surgery. Active and active-assisted exercises were initiated after the immobilization period. After 6 weeks, patients initiated strengthening exercises of the rotator cuff and scapular stabilizers. Rehabilitation was performed at least 3 months after surgery with the assistance of a physical therapist. Full return to sports or heavy labor was allowed after 6 months.

### 2.4 Statistical analysis

A paired or unpaired *t*-test were used to compare differences in each outcome between the baseline and 12 months after ARCR as appropriate. Fisher's exact test was used for categorical variables. Statistical significance was set at p < 0.05. All analyses were conducted using SPSS® v25.0 (IBM Inc., Armonk, NY, USA).

## 3 Results

Demographic characteristics of patients are presented in Table 1. Demographic data were not significantly different between the Low and High groups. Preoperative ROM, Constant Shoulder Score, and UCLA scores showed significant improvement at 12 months post-ARCR in both groups (p < 0.05) except external rotation in High group (Table 2). Further, forward flexion, Constant Shoulder Score, and UCLA score were significantly inferior in the High group compared to the Low group (Low/High group; 167.3 ± 7.7°/159.5 ± 16.3° for forward flexion; p = 0.013, 94.9 ± 7.6/90.1 ± 9.9 points for Constant Shoulder Score; p = 0.033, 32.6 ± 3.6/30.6 ± 4.1 points for UCLA score, p = 0.037). Moreover, HbA1c levels at 12 months post-ARCR were also significantly higher in the High group compared to the Low group (Low/High group; 6.5 ± 0.3%/7.6 ± 0.8%; p < 0.05) (Table 3). The rate of rotator cuff retear was not significantly different between groups (Sugaya type4; p = 0.49, type5; p = 0.322), and no cases of infection or shoulder stiffness were noted in either group (Table 4).

**Table 1:** Patient's demographics at baseline.

Variables	High group	Low group	P-value
Number of shoulders	25	26	
Male/Female	16/9	13/13	0.4
Age (years)	63.7 ± 9.4	64.7 ± 9.8	0.35
Duration of symptoms before ARCR (months)	6.3 ± 3.9	9.4 ± 11.5	0.15
Affected side (right/left)	16/9	15/11	0.367
HbA1c (%)	7.5 ± 0.8	7 ± 1.1	0.03∗
Stiff shoulder (n)	3	3	0.959
Preoperative findings			
ROM			
forward flexion (°)	128.3 ± 39.6	123.4 ± 33.6	0.322
external rotation (°)	52.7 ± 12.7	47.8 ± 16.3	0.124
internal rotation (point)	3.3 ± 2.2	2.6 ± 2.3	0.164
Constant Shoulder Score (point)	60.6 ± 15	59.1 ± 10.5	0.343
UCLA scores (point)	18.7 ± 5.4	18.3 ± 4.1	0.374

**Table 2:** High group.

High group			
Variables	Pre	Po12 months	P-value
ROM			
forward flexion (°)	128.3 ± 39.6	159.5 ± 16.3	<0.05∗
external rotation (°)	52.7 ± 12.7	54.7 ± 14.6	0.417
internal rotation (point)	3.3 ± 2.2	4.6 ± 1.3	<0.05∗
Constant Shoulder Score (point)	60.6 ± 15	90.1 ± 9.9	<0.05∗
UCLA scores (point)	18.7 ± 5.4	30.6 ± 4.1	<0.05∗
Low group			
ROM			
forward flexion (°)	123.4 ± 33.6	167.3 ± 7.7	<0.05∗
external rotation (°)	47.8 ± 16.3	57.6 ± 6.9	<0.05∗
internal rotation (point)	2.6 ± 2.3	5.2 ± 1	<0.05∗
Constant Shoulder Score (point)	59.1 ± 10.5	94.9 ± 7.6	<0.05∗
UCLA scores (point)	18.3 ± 4.1	32.6 ± 3.6	<0.05∗

**Table 3:** 12 months after ARCR.

Variables	High group	Low group	P-value
ROM			
forward flexion (°)	159.5 ± 16.3	167.3 ± 7.7	0.013∗
external rotation (°)	54.7 ± 14.6	57.6 ± 6.9	0.151
internal rotation (point)	4.6 ± 1.3	5.2 ± 1	0.084
Constant Shoulder Score (point)	90.1 ± 9.9	94.9 ± 7.6	0.034∗
UCLA scores (point)	30.6 ± 4.1	32.6 ± 3.6	0.037∗
HbA1c (%)	7.6 ± 0.8	6.5 ± 0.3	<0.05∗

**Table 4:** Complications at 12 months after ARCR.

Variables	High group	Low group	P-value
Rotator cuff retear			
Sugaya type 4	1	0	0.49
Sugaya type 5	0	1	0.322
Infection	0	0	0
Shoulder stiffness	0	0	0

## 4 Discussion

This study shows that clinical outcomes of diabetic patients who undergo ARCR and have poor postoperative glycemic control are significantly inferior to those patients with good glycemic control. Contrastingly, the results revealed that there is no association between postoperative glycemic control and the incidence of complications following ARCR.

Diabetes mellitus has been cited as a risk factor for postoperative shoulder stiffness, infection, and decreased bone-tendon healing rates.^3^^,^^6^^,^^14^, ^15^, ^16^^,^^21^, ^22^, ^23^, ^24^Koorevaar et al.^23^reported that diabetes mellitus as a significant predictor of postoperative shoulder stiffness. In contrast, Chung et al.^24^ and Cho et al.^6^ reported that no association between the presence of diabetes and postoperative shoulder stiffness after ARCR. Regarding infection, Borton et al.^15^ and Clement et al.^14^ found no significant differences between diabetic and nondiabetic patients that underwent ARCR. In contrast, Jourdan et al.^3^ reported that patients with perioperative HbA1c levels ≥8.0 mg/dL had significantly increased postoperative infection rates compared with those with levels below that threshold value. Regarding the rate of rotator cuff retear and revision surgery, Jourdan et al.^3^ and Clement et al.^14^ reported no association between perioperative glycemic control and the incidence of revision surgery. Miyatake et al.^16^ reported that there was not a significant difference in the retear rate between diabetic and nondiabetic patients who underwent ARCR. On the contrary, Borton et al.^15^ reported that diabetic patients were almost twice as likely to experience cuff retears compared to nondiabetic patients (26.3% vs. 15.6%, p = 0.042).

Only a few studies have compared clinical outcomes after ARCR in controlled and uncontrolled diabetic patients.^6^^,^^13^^,^^16^ Cho et al.^6^ reported that the retear rate (43%) in uncontrolled diabetic patients (preoperative serum HbA1c ≥ 7%) was significantly higher than in controlled diabetic patients (26%) (preoperative serum HbA1c < 7%). Cho et al. concluded that sustained hyperglycemia increased the possibility of anatomical failure of the repaired rotator cuff. In contrast, Miyatake et al.^16^ reported that the clinical and structural outcomes after ARCR in diabetic patients with perioperative glycemic control were comparable to those in non-diabetic patients. The study did not show any significant differences in retear rates between the diabetic and non-diabetic patients or between controlled (preoperative serum HbA1c < 7%) and uncontrolled (preoperative serum HbA1c ≥ 7%) diabetic patients. The difference between the two studies is that uncontrolled diabetic patients were admitted to the hospital before surgery. In Miyatake's report, uncontrolled diabetic patients were admitted to the hospital before surgery to undergo intensive glycemic control and education. Therefore, there were far fewer patients with poor glycemic control perioperatively compared with those in the study by Cho et al. Similarly, our previous study^13^ reported that diabetic patients with poor glycemic control were preoperatively hospitalized for perioperative diabetic control and there were no significant differences in clinical outcomes and complications after ARCR between controlled and uncontrolled diabetic patients.

The current study shows that diabetic patients with poor glycemic control postoperatively had significantly inferior clinical outcomes after ARCR compared to those with good glycemic control. Although previous studies demonstrated that perioperative hyperglycemia has adverse effects after ARCR,^3^^,^^15^^,^^25^ the results of the current study suggest that intensive glycemic control, perioperatively and postoperatively, is imperative in the prevention of complications and optimization of good clinical outcomes after ARCR.

Our study has several limitations. First, the study design was retrospective. Second, there was a relatively small sample size and short follow-up period. Third, the definition of uncontrolled diabetic patients with poor glycemic control (preoperative serum HbA1c ≥ 7%) was somewhat arbitrary. However, the criteria to quantify the severity of sustained hyperglycemia in diabetic patients remains ambiguous even after consulting with endocrinological specialists.

In conclusion, patients with diabetes showed improvement of ROM and function after ARCR; however, the improvements in diabetic patients with postoperative poor glycemic control were significantly inferior. Intensive glycemic control should be recommended for diabetic patients not only perioperatively but also postoperatively.

## Funding

None.

## Declaration of competing interest

None.

## List of abbreviations

ARCR: arthroscopic rotator cuff repair
HbA1c: hemoglobin A1c
MRI: magnetic resonance imaging
ROM: range-of-motion
UCLA: University of California, Los Angeles
